# Will the advances in retrograde intrarenal surgery extinguish percutaneous nephrolithotomy for stones larger than 2 cm?

**DOI:** 10.1590/S1677-5538.IBJU.2022.0533

**Published:** 2022-11-20

**Authors:** Wilmar Azal, Lukas Costa de Salles, Bruno di Domenico, Ricardo Miyaoka, Leonardo O. Reis

**Affiliations:** 1 Universidade Estadual de Campinas Campinas SP Brasil Universidade Estadual de Campinas (Unicamp), Campinas, SP, Brasil; 2 Pontifícia Universidade Católica de Campinas Campinas SP Brasil UroScience, Pontifícia Universidade Católica de Campinas (PUC-Campinas), Campinas, SP, Brasil

## COMMENT

Since 1976, when percutaneous nephrolithotomy (PCNL) was first described (1), it has been an excellent choice of endourological treatment for large renal stones. In fact, both American Urological Association (AUA) and European Association of Urology (EAU) guidelines currently consider PCNL the preferred surgical approach to stones larger than 2 cm (2, 3). However, recent technological advances in retrograde intrarenal surgery (RIRS) including development of digital ureteroscopes and high-power lithotripsy generators result in better stone free rates (SFR) while offering less morbidity to patients, hence broadening the indications of this technique, including large and complex stones (4, 5). Better outcomes translate into an increasing number of RIRS worldwide (6), but bring about the question: will RIRS, ultimately, extinguish PCNL as the main surgical treatment for large stones?

The key gamechangers related to RIRS evolution include the development of disposable ureteroscopes and the new Thulium fiber laser (TFL). Single-use devices offer some advantages over the reusable flexible ureteroscopes: they are lighter (which may prevent fatigue in long lasting cases especially when treating large burden stones), offer a better deflection angle and provide superior image quality (7). Other authors also observed single-use device was associated with shorter operative time and higher stone free rates with possibly less complications (8–10). Moreover, the use of disposable material may reduce total costs to the health care system, which is vital within developing countries perspectives, including Brazil (7). Current literature on TFL provides compelling results when compared to Holmium laser, indicating it is a milestone in RIRS: higher stone ablation rate (2 to 4 times faster), less calculi retropulsion and more efficient fragmentation generating smaller fragments. The possibility of using laser fibers as thin as 150 micrometers can provide better scope deflection (11) and could allow for future further instruments miniaturization (12–14).

Nevertheless, complications associated with RIRS cannot be underestimated – not only because of its continuously increasing use but also because of their potential severity (15). Ureteral access sheath (UAS) facilitates fragment basketing if the surgeon opts for stone fragmentation and provides a better irrigating flow – which is essential for better visualization and maintenance of pelvicalyceal temperature and low pressure and therefore might play an active role in the procedure success (16–18). However, Traxer et al. reported on an overall incidence of UAS related ureteral lesions of 46.5%, of which 13.3% were classified as severe (19). This can develop into both short (such as hematuria and the need of ureteral stent for an extensive period) and long-term complications (such as ureteral stenosis) and should be avoided.

Furthermore, a recent publication showed that UAS increased the odds of a post-operative emergency department visit and re-hospitalization, without better SFR (18). Unusual but dramatic complications related to the UAS have been described, such as the entrapment of a flexible ureteroscope (fURS) inside the sheath due to a breakage of the outer surface of the scope caused by excessive manipulation (20).

Rise in intra-renal temperature during stone fragmentation is another concern in RIRS, and it is related to high laser power (21), prolonged time of pedal activation and irrigation pressure. They may implicate in fluid heating and thermal dose exposition (22). An in vitro model with UAS and common Holmium laser settings verified high temperatures can result after as little as 1 second of laser activation especially at power settings over 10 W (23).

Another key aspect of RIRS procedure is the use of ureteral double J stents before or following the procedure. A meta-analysis from Chang et al. concluded that pre-stenting may improve stone free rates in fURS for large kidney stones, with no difference in complication rate (24).

A study that analyzed almost 10,000 ureteroscopies, observed 73% of ureteral stenting following surgery. Pre-stented status, age, stone size and location were associated with stent use after surgery. Stent usage significantly increased the odds of an unplanned emergency hospital visit after surgery (25).

Also, “forgotten stent” can develop into severe encrustation (26) and its removal may require refined management planning and advanced surgical techniques (27, 28). Strategies to prevent such problem include stent judicious use and the implementation of modern technology to keep track of stented patients (29, 30).

There are potentially life-threatening complications in RIRS even in experienced endourologists hands (31). A systematic review showed an incidence of 0.45% of post RIRS perirenal hematoma with a mean stone size of 1.7 cm, and in which 17.5% of the patients needed surgical intervention – resulting eventually in nephrectomy and even death (4).

Furthermore, longer surgical time had a significant association with systemic inflammatory response syndrome (SIRS) and urosepsis after fURS, which occurred in 6.9% and 5.0%, respectively, among over 8.000 studied patients (32, 33). Other reports reported on a rise in RIRS-related deaths over the past decade, associating high stone burden as predictive factor for worse results, requiring an effort to reduce operative time with staged procedures if needed in order to decrease morbidity, rehospitalization, and mortality following ureteroscopies (15, 34).

Residual fragments after RIRS also merits attention. Sur et al. reported on 20-43% of residual fragments are associated with stone events including pain and emergency department visits, reinterventions, and even calculi regrowth (35). A series of fURS comprising more than 400 patients with stones larger than 2 cm revealed a cumulative SFR of 85% (36). However, in nearly all cases, plain abdominal radiograph and/or renal ultrasound were used to assess residual fragments, possibly leading to an over-estimation of the clearance result. Indeed, when using computerized tomography (CT) to determine SFR, results are less than satisfying. Studies which performed abdominal CT scan up to 3 months after initial RIRS showed visible residual fragments varying from 38 to 50% of the procedures (37, 38). Portis et al. prospectively evaluated patients with renal calculi up to 15 mm, and even after a special effort to clear all stones in fURS (by using ureteral sheaths, breaking the stones in upper pole and actively retrieving all fragments), achieved a complete removal status by CT criteria in only 54% of cases (39).

One could argue that residual fragments smaller than 4 mm are less likely to experience post-operative stone growth, complications or require reintervention (5). Rebuck et al. reported a 19.5% chance of experiencing a calculus related event (such as emergency visit, hospitalization or surgery) after RIRS in patients with post-operatory fragments up to 4 mm by CT measurement (40). In fact, according to a review about the natural history of asymptomatic residual stone after this procedure, there was a 44% chance of a stone related event: re-intervention was predictable based on fragment size (p=0.017), calculi < 4 mm led to 18% re-operation (vs. 38% in > 4 mm), and even residual stones > 2 mm were significantly likely to grow (41).

On the other hand, not only RIRS has evolved, but PCNL has also been fighting its way to remain an attractive option for treating large stones. In fact, when analyzing stone procedures, while the proportion of PCNLs has remained fairly stable over the last years, the number of urologists performing their own percutaneous access instead of delegating it to an interventional radiologist has increased substantially (42). Moreover, there are accumulating publications on the development and advantages of ultrasound-guided renal puncture which reinforces the interest of the scientific community on this (43). Ultrasound may offer significant clinical gains for PCNL execution. Lin et al. described identification of a fused renal pyramid by US and doppler use to identify ectopic blood vessels in order to reduce bleeding during calycinal access in percutaneous surgery (44). Moreover, US guidance provides visualization of adjacent viscera, delineation of anterior and posterior calyces, reduction of radiation exposure, real-time imaging of renal parenchyma and detection of radiolucent stones (45).

But perhaps, the most notorious evolution in standard PCNL was the significant shift to miniaturized PCNL (mini-PCNL) allowing reduced parenchymal renal injury. This technique offers a midway option between conventional PCNL and less invasive endoscopic procedures such as RIRS and implicates in using a tract smaller than 22F (46). The reusable equipment and the vacuum cleaner “vortex” effect make mini PCNL more affordable than standard PCNL. Dilation can be performed either in one-shot or with a progressive technique; and the possibility to enlarge from a small to a thicker tract if needed (Matrioska technique) presents mini-PCNL as a very versatile strategy, suitable for the treatment of almost any stone, including those larger than 2 cm (47). When comparing bleeding, a prospective randomized controlled trial reported that mini-PCNL had a significantly lower drop in hematocrit level versus standard PCNL (p=0.02) and less pain at 6 and 24 hours after surgery (48).

Research in new technologies aiming to improve PCNL outcomes continue to blossom. While the high-power lasers can also be used in percutaneous procedures, other lithotripters specific for this surgery have been created. A prospective comparative study of mini-PCNL using Trilogy lithotripter versus TFL in renal stones with a mean size > 2 cm showed that Trilogy achieved significantly better stone fragmentation rate (49). Regarding better learning of renal anatomy and PCNL technique, Parkhomenko et al. described the use of an immersive virtual reality renal model (50). Likewise, Keyu et al. developed a “3D printing personalized percutaneous nephrolithotomy guide plate for PCNL” which allowed for reduced intra-operative blood loss and bleeding related complications (51).

Less aggressive percutaneous procedures led to the proposal of day-hospital discharge. A systematic review from the European Society observed that, for selected patients, standard PCNL is safe and efficient with a low rate of complications or readmissions (52). A propensity score-matching study evaluating day-cases versus inpatient mini-PCNL concluded that the same day discharge PCNL was more cost-effective, with no significant difference in complications along with very low unplanned readmission during the postoperative period of 14 days (53). And a multi-institutional experience compared micro-PCNL in a group of patients who also had same-day discharge versus an inpatient group and reported on equivalent SFR and complication rate (54).

Finally, it is known that RIRS by itself does not offer full access to reach all renal calculi, especially those in lower calyx with long and narrow infundibulum (55). Karim et al. published a systematic review where they concluded that steep infundibular pelvic angle (IPA) (< 30°) seems to be the most important predictor for failure in the treatment of lower pole stones using RIRS, followed by operative time duration and large calculi burden (56). Inoue et al. also showed that an IPA <30° was the only negative risk factor for stone clearance after flexible ureteroscopy for large renal stones (>15 mm) according to their multivariate analysis (57). Tastemur et al. observed that stone size and IPA (< 42.6°) were independent risk factors for success of RIRS procedure (58).

Ozimek et al. analyzed almost 400 RIRS and reported on that steep IPA could be considered the first risk factor predictor for both flexible ureteroscope damage and significant unfavorable postoperative course – occurrence of complications Clavien-Dindo 2 as well as prolonged hospital stay (59). A meta-analysis comparing mini-PCNL and RIRS for the treatment of lower pole stones up to 2 cm reported similar operative and fluoroscopy times, complication rates and length of hospital stay, although mini-PCNL was significantly superior in terms of success rate (60). A recent publication proposed a scoring system based on pre-operative exams and SFR for better selection of endoscopic treatment for lower pole renal stones. A score was given after analyzing the IPA and stone number and diameter, and infundibular length and width, ultimately providing guidance for urologists to decide upon retrograde or percutaneous access (61).

Overall, systematic reviews and meta-analysis comparing directly RIRS and PCNL for renal stones > 2 cm suggest balancing risks and benefits and tailor an individual treatment strategy in a patient-doctor sharing decision (62, 63). Also, previous standard percutaneous nephrolithotomy might impair retrograde intrarenal surgery outcomes (64). However, not only the RIRS and PCNL are not to be seen as competitors, but possibly as complementary - so that endoscopically combined intrarenal surgery (ECIRS) has opportunistically emerged and set its place as another gamechanger. PCNL (and its miniaturizations) will definitely not be extinguished, as both retrograde and percutaneous accesses keep evolving and safer and more efficient procedures develop.
